# Routine Cerebrospinal Fluid (CSF) Parameters in Patients With Spinal Muscular Atrophy (SMA) Treated With Nusinersen

**DOI:** 10.3389/fneur.2019.01179

**Published:** 2019-11-07

**Authors:** Claudia D. Wurster, Jan C. Koch, Isabell Cordts, Jens Dreyhaupt, Markus Otto, Zeljko Uzelac, Simon Witzel, Benedikt Winter, Tugrul Kocak, Michael Schocke, Patrick Weydt, Kurt Wollinsky, Albert C. Ludolph, Marcus Deschauer, Paul Lingor, Hayrettin Tumani, Andreas Hermann, René Günther

**Affiliations:** ^1^Department of Neurology, Ulm University, Ulm, Germany; ^2^Department of Neurology, University Medicine Göttingen, Göttingen, Germany; ^3^Department of Neurology, Klinikum Rechts der Isar der Technischen Universität München, Munich, Germany; ^4^Institute of Epidemiology and Medical Biometry, Ulm University, Ulm, Germany; ^5^Department of Pediatrics, Ulm University, Ulm, Germany; ^6^Department of Orthopedic Surgery, RKU - University and Rehabilitation Clinics, Ulm University, Ulm, Germany; ^7^Department of Neuroradiology, RKU - University and Rehabilitation Clinics, Ulm University, Ulm, Germany; ^8^Department for Neurodegenerative Disorders and Gerontopsychiatry, Bonn University, Bonn, Germany; ^9^Department of Anesthesiology, RKU - University and Rehabilitation Clinics, Ulm University, Ulm, Germany; ^10^German Center for Neurodegenerative Diseases (DZNE) Ulm, Ulm, Germany; ^11^Specialty Hospital of Neurology Dietenbronn, Schwendi, Germany; ^12^Translational Neurodegeneration Section “Albrecht-Kossel”, Department of Neurology, University Medical Center Rostock, University of Rostock, Rostock, Germany; ^13^German Center for Neurodegenerative Diseases (DZNE) Rostock, Rostock, Germany; ^14^Department of Neurology, Technische Universität Dresden, Dresden, Germany; ^15^German Center for Neurodegenerative Diseases (DZNE) Dresden, Dresden, Germany

**Keywords:** spinal muscular atrophy, motor neuron disease, antisense-oligonucleotide, routine CSF parameters, lumbar puncture

## Abstract

**Background:** Nusinersen is an antisense-oligonucleotide (ASO) approved for treatment of 5q-spinal muscular atrophy (SMA). Since the drug cannot cross the blood-brain barrier (BBB), it must be administered into the cerebrospinal fluid (CSF) space repeatedly by lumbar puncture. However, little is known whether ASOs have an impact on CSF routine parameters that may yield information on CSF flow and/or intrathecal inflammation. The objective of this study was to examine CSF routine parameters in SMA patients treated with nusinersen.

**Methods:** Routine CSF parameters [white cell count, total protein, CSF/serum quotients of albumin (Qalb), lactate, and oligoclonal IgG bands (OCB)] of 60 SMA patients (type 1, 2, and 3, aged 7–60 years) were retrospectively analyzed.

**Results:** White cells ranged from 0 to 4/μL in CSF; a singular case of pleocytosis (8/μL) was observed in a patient in parallel with a systemic infection. Total protein and Qalb showed a mild increase from baseline to the following lumbar punctures (except for total protein in CSF at the fourth injection of nusinersen). Lactate levels revealed a stable course. In one patient, positive OCB in CSF were transiently observed. The slight change in total CSF protein and Qalb may be caused by repeated lumbar puncture and/or intrathecal administration of the drug.

**Conclusion:** Our data suggest that a regular examination of routine CSF parameters in patients in which intrathecal ASOs are administered is important to obtain information on possible side effects and to gain further insights into intrathecal processes.

## Introduction

Spinal muscular atrophy (SMA) is a rare neuromuscular disease caused by homozygous deletion or point mutation in the survival of motoneuron (*SMN*) 1 gene on chromosome 5 leading to muscle weakness and muscle atrophy (1). Nusinersen is an antisense-oligonucleotide (ASO) approved for treatment of 5q-associated SMA (2). ASOs are synthetic single-strand nucleic acid sequences that selectively bind to nucleotide sequences of RNA-strands and alter protein synthesis by different mechanisms of action. Thus, ASOs provide a novel opportunity to address previously inaccessible drug targets (3, 4). Since the drug cannot cross the blood-brain barrier (BBB), it must be administered repeatedly into the cerebrospinal fluid (CSF) space by lumbar puncture. In July 2018, the appearance of communicating hydrocephalus not related to meningitis or bleeding has been reported in five patients treated with nusinersen (5, 6); it still remains unclear if these events are directly related to the drug. Moreover, three cases of drug-induced aseptic meningitis (DIAM) were reported recently (6). Since there is limited experience in clinical practice with ASOs so far, not much is known whether ASOs have an impact on blood-CSF barrier (BCB) function or CSF flow [assessed by albumin CSF/serum quotient (Qalb)] (7) and on other CSF routine parameters. In a phase 1 study to examine safety, tolerability, pharmacokinetics, and preliminary clinical efficacy of nusinersen no significant changes in CSF parameters (cell counts, protein, and glucose) have been observed. However, we saw a slight increase of total protein in CSF and Qalb after four injections of nusinersen in a previous study (8). CSF is a dynamic and metabolically active fluid surrounding the central nervous system (CNS). Diagnostic evaluation of inflammatory (infectious or non-infectious) or degenerative conditions, involving the brain, spinal cord, and meninges, is possible by combining a set of values referred to as CSF routine parameters (9). The objective of this study was to examine CSF routine parameters [white cells, total protein, Qalb, lactate, and oligoclonal IgG bands (OCB)] in 60 SMA patients (type 1, 2, and 3, aged 7–60 years) treated with nusinersen.

## Materials and Methods

### Standard Protocol Approvals, Registrations, and Patient Consents

The study was approved by the local ethics committees of the centers involved in Ulm, Dresden, Göttingen, and Munich (approval number at central study center at the University of Ulm 19/12; 2012) and all patients or their relatives (legal guardian) gave informed written consent to participate in the study.

### Participants and Sampling

Patient samples were collected from June 2017 to April 2019 at the following centers of the MND-NET (German Network for Motoneuron Diseases): Department of Neurology, Ulm University Hospital (Germany), Department of Neurology, Technische Universität Dresden (Germany), Department of Neurology, University Medical Center Göttingen (Germany) and Department of Neurology, Klinikum rechts der Isar der Technischen Universität München (Munich, Germany).

All patients had genetically confirmed 5q-associated SMA (deletion in exon 7 and/or 8 in the *SMN1* gene). Patients history and clinical data were gathered before therapy started. Motor scores [including Children's Hospital of Philadelphia Infant Test of Neuromuscular Disorders (CHOP INTEND) (10) in SMA type 1 patients and Hammersmith Functional Motor Scale-Expanded (HFMSE) (11) in SMA type 2 and 3 patients] were performed on the same days as the administration of nusinersen (except on treatment days 14 and 28).

CSF and serum samples were taken before the intrathecal administration of nusinersen on treatment days 0 (T1), 14 (T2), 28 (T3), 63 (T4), 180 (T5), 300 (T6), 420 (T7), and 540 (T8). CSF was obtained in all patients by lumbar puncture; no intracisternal or intracervical puncture was performed in any patient.

The following CSF parameters were determined in all centers: white cell count, total protein, Qalb, CSF/serum quotient of IgG, IgA, IgM, and lactate. Glucose were only measured in two centers. OCB of SMA samples were regularly assessed in four of five centers. Erythrocytes were determined in all centers, but with different reporting standards (qualitatively and/or quantitatively). Cytological examination of CSF was also carried out if required. CSF opening pressure was not routinely recorded by the centers; the main reason for this was the difficulty to place patients in a lateral position (especially in CT-guided lumbar punctures which were often performed in prone position). Of these CSF parameters, white cells, total protein, lactate, Qalb, and OCB were selected for systematic analysis for this study.

### Sample Analysis

CSF was collected in polypropylene tubes and routine CSF parameters were immediately analyzed. Determination of white cell count was performed microscopically in a Fuchs-Rosenthal chamber (12). Total protein and albumin were measured by standard nephelometry in CSF and serum (Dade-Behring nephelometer analyzer, Marburg, Germany) using a polyclonal antibody in the case of albumin as described earlier (13). Qalb was included as a measure of the BCB function and CSF flow, respectively (7, 14). As the Qalb is age dependent, patients with $Qalb>\left(4+\;\frac{Age\;at\;LP}{15}\right)*10^{-3}$ were considered to have a dysfunction of the BCB or rather CSF flow. CSF lactate was determined by a lactate-oxidase reaction (Greiner GmbH, Flacht, Germany). Detection of OCB was performed by isoelectric focusing (center-dependent) on agarose or polyacrylamide gels and subsequent immunoblotting or immunofixation using an IgG-specific antibody staining (Servalyte, Serva) (15). OCB were characterized according to the following criteria: no oligoclonal IgG bands in CSF (OCB type 1), oligoclonal IgG bands in CSF, but not in serum (OCB type 2), oligoclonal IgG bands in CSF and serum with additional oligoclonal IgG bands in CSF (OCB type 3), identical oligoclonal IgG bands in CSF and serum (OCB type 4), and monoclonal IgG bands in CSF and serum (OCB type 5). Thus, OCB were considered positive (intrathecally produced) if patterns 2 or 3 were present (16). CSF samples with massive contamination of erythrocytes were excluded to avoid false positive cell count, total protein, Qalb, and lactate values. This definition included all samples in which CSF was described as macroscopically “bloody” and/or in which “mass presence of erythrocytes” was observed.

### Statistical Analysis

Continuous variables were described by the mean and standard deviation or median and quartiles as appropriate. Additionally, the range is presented. Categorical variables were described with absolute and relative frequencies. OCB were indicated according to their pattern (type 1–5) and are depicted descriptively. To compare datasets of marker concentrations within the SMA patient group, the Wilcoxon signed rank test was used. Associations between marker concentrations and demographic and clinical characteristics were investigated by the Spearman rank correlation coefficient (rho). The point-biserial correlation coefficient (rPB) was used in case of a continuous and a binary variable. Because of the explorative nature of this study, the results of the statistical tests should not be interpreted as confirmatory: all results of statistical tests have to be interpreted as hypothesis-generating only. No adjustment for multiple testing was done. A two-sided *p*-value ≤ 0.05 was interpreted as statistically significant. Statistical analysis was performed with the software GraphPad Prism 8 and SAS 9.4 under Windows.

## Results

### Patient and Sample Characteristics

For a summary of demographic patient characteristics see Table 1.

**Table 1:** Summary of demographic patient characteristics.

	**SMA**	**SMA 1**	**SMA 2**	**SMA 3**
*N*	60	2	28	30
Gender (% males)	55	50	46	63
Age (y)	31.9 (7–60)	7; 15	26.1 (11–48)	38.7 (15–60)
HFMSE			2 (0–8)	24 (0–66)
CHOP INTEND		22; 26		
LP (% CT-guided)	65	50	100	33
Spinal hardware	19	1	16	2
Severe scoliosis	18	0	11	7
Obesity^*^	2	0	1	1

Age in mean (range); HFMSE in median (range). SMA, spinal muscular atrophy; N, number of patients; y, years; HFMSE, Hammersmith Functional Rating Scale Expanded; CHOP INTEND, Children‘s Hospital of Philadelphia Infant Test of Neuromuscular Disorders; LP, lumbar puncture.

* *Marked obesity making conventional LP impossible*.

In total, CSF of 63 SMA patients was collected; CSF samples of 3 patients were excluded from analysis due to massive contamination of erythrocytes in baseline CSF (T1), thus analysis included CSF of 60 patients. Two samples of one patient were excluded in the following (CSF of T6 and T7) because of massive contamination of erythrocytes during lumbar puncture T6, which was performed intraoperatively during change of growing rods. Another sample of a second patient was excluded from analysis at T5 due to massive contamination of erythrocytes caused by a traumatic lumbar puncture.

Of the 60 patients, 9 patients received 8, 17 patients received 7, 12 received 6, 10 received 5, and 12 received 4 injections of nusinersen. In these 60 patients, in total 361 successful lumbar punctures were performed. CSF of five lumbar punctures was not available for analysis for different reasons (e.g., too little CSF volume). CSF analyses of 40 lumbar punctures were incomplete (with regard to the selected parameters white cells, total protein, Qalb, lactate and OCB). In baseline CSF (T1) white cell count, total protein and lactate were analyzed in all patients, Qalb in 57 patients and OCB in 51 patients.

### Analysis of Routine CSF Parameters

Median values with quartiles and ranges of white cell count, total protein, Qalb, lactate, and number of samples with positive OCB in CSF (type 2 and 3) at treatment days T1–T8 are shown in Table 2.

**Table 2:** Illustration of routine CSF parameters in SMA patients undergoing treatment with nusinersen.

	**White cells (count/μL)**	**Total protein (mg/L)**	**Qalb (×10^**3**^)**	**Lactate (mmol/L)**	**OCB**
T1	1.0 [0.0;1.8] range 0.0–4.0 *N* = 60	319.5 [246.8;421.0] range 148.0–715.0 *N* = 60	4.20 [3.45; 6.35] range 2.20–11.40 *N* = 57	1.40 [1.30;1.60] range 0.90–2.80 *N* = 60	Positive in CSF (type 2/3): 2 *N* = 51
T2	1.0 [1.0; 2.0] range 0.0–4.0 *N* = 59	343.0 [268.0; 422.0] range 153.0–1300.0 *N* = 59	4.70 [3.73; 6.40] range 2.30–15.60 *N* = 56	1.40 [1.25; 1.61] range 1.00–2.80 *N* = 58	Positive in CSF (type 2/3): 2 *N* = 51
T3	1.0 [0.0; 2.0] range 0.0–8.0 *N* = 58	339.0 [275.5; 449.3] range 139.0–1110.0 *N* = 56	4.57 [3.80; 6.55] range 2.19–14.34 *N* = 51	1.44 [1.30; 1.60] range 1.08–3.05 *N* = 57	Positive in CSF (type 2/3): 3 *N* = 52
T4	1.0 [0.0; 1.0] range 0.0–4.0 *N* = 58	318.0 [268.3; 437.3] range 178.0–1040.0 *N* = 56	4.70 [3.68; 6.94] range 2.60–15.00 *N* = 50	1.40 [1.23; 1.60] range 1.03–2.80 *N* = 57	Positive in CSF (type 2/3): 2 *N* = 49
T5	1.0 [0.0; 2.0] range 0.0–4.0 *N* = 46	352.0 [280.8; 439.0] range 184.0–809.0 *N* = 46	4.70 [3.75; 6.16] range 2.14–11.20 *N* = 45	1.50 [1.27; 1.70] range 1.00–3.00 *N* = 45	Positive in CSF (type 2/3): 2 *N* = 43
T6	1.0 [0.5; 2.0] range 0.0–3.0 *N* = 37	347.0 [292.5; 464.5] range 175.0–809.0 *N* = 37	4.90 [3.88; 6.37] range 2.42–13.14 *N* = 37	1.45 [1.23; 1.70] range 1.00–3.10 *N* = 37	Positive in CSF (type 2/3): 1 *N* = 37
T7	1.0 [1.0; 1.0] range 0.0–3.0 *N* = 25	355.0 [302.0; 438.5] range 172.0–838.0 *N* = 25	5.00 [3.85; 6.63] range 2.39–12.48 *N* = 25	1.40 [1.21; 1.70] range 1.07–2.30 *N* = 25	Positive in CSF: type 2/3: 0 *N* = 25
T8	1.0 [1.0; 2.0] range 0.0–4.0 *N* = 9	322.0 [282.0; 433.5] range 218.0–592.0 *N* = 9	4.15 [3.65; 6.83] range 3.50–7.81 *N* = 8	1.30 [1.20; 1.57] range 1.13–1.90 *N* = 9	Positive in CSF: type 2/3: 0 *N* = 5

*Median values with quartiles (in square brackets) and ranges of white cell count, total protein, and lactate in CSF and of Qalb at treatment days T1–T8 are shown; number of samples with positive IgG bands in CSF (OCB type 2 and 3) at treatment days T1–T8 is presented. Qalb, albumin CSF/serum quotient; OCB, oligoclonal IgG bands; N, number of available samples*.

#### White Cell Count

Apart from a single case of CSF pleocytosis, white cells in CSF ranged from 0 to 4/μL. In CSF of an 18-year-old female SMA type 2 patient, we observed an increased cell count of 8/μL (activated lymphomonocytic cells with isolated hemosiderophages) at T3. The patient reported from symptoms of gastrointestinal infection along with back pain and headache and presented further signs of meningeal irritation (e.g., positive Lasègue sign, nausea and vomiting) 1 week before the lumbar puncture at T3; MRI of the neurocranium and the lumbar spine revealed no pathological findings at this time (1 week before T3). Of note, cell count in CSF of this patient was in the normal range again (1/μL) at T4.

Although there was a statistical difference between the white cell count of T1 and T2 (*p* = 0.0035), analysis of white cell count showed stability over time [T1–T3 (*p* = 0.0570), T1–T4 (*p* = 0.5556), T1–T5 (*p* = 0.9236), T1–T6 (*p* = 0.8069), T1–T7 (*p* = 0.8750), and T1–T8 (*p* = 0.3750)].

#### Total Protein

Twelve SMA patients showed slightly elevated total protein levels above the reference boarder of 450 mg/L at baseline (range 465–715 mg/L). In the remaining 48 patients, total protein levels were detected below the reverence boarder of 450 mg/L in baseline CSF.

CSF total protein differed significantly from baseline (T1) to all following lumbar punctures [T1–T2 (*p* = 0.0004), T1–T3 (*p* < 0.0001), T1–T4 (*p* = 0.0012), T1–T5 (*p* < 0.0001), T1–T6 (*p* = 0.0001), T1–T7 (*p* = 0.0010), and T1–T8 (*p* = 0.0195)]. While a mild increase in total protein could be observed from baseline (T1) to treatment days T2, T3, T5, T6, T7, and T8, median total protein levels were slightly lower at T4 compared to baseline protein levels.

The highest total protein levels of all CSF samples [1300 mg/L (T2), 1110 mg/L (T3), 1040 mg/L (T4)] were detected in a 46-year-old SMA type 3 patient with baseline total protein of 710 mg/L (T1), who showed the highest (age-related) Qalb levels of all samples in accordance [10.4 × 10^3^ (T1), 15.6 × 10^3^ (T2), not analyzed (T3), 15.0 × 10^3^ (T4)]; white cell count in this patient was within the normal range, lactate levels minimal elevated at T4 (2.2 mmol/L) and OCB were not determined. Apart from a pronounced scoliosis, there were no further anamnestic or clinical abnormalities in this patient, neither before nor during therapy. The highest total protein (715 mg/L) along with the highest (age-related) Qalb level (11.4 × 10^3^) in baseline CSF (T1) was detected in a 15-year-old SMA type 3 patient, in whom positive OCB in CSF (type 2) in baseline and follow-up samples were observed. In this patient, total protein slightly increased to 913 mg/L in the second lumbar puncture (T2), but showed a stable course in the following lumbar punctures [761 mg/L (T3), 791 mg/L (T4), 728 mg/L (T5); Qalb levels showed a similar performance (12.2 × 10^3^ (T2), 11.0 × 10^3^ (T3), 11.7 × 10^3^ (T4), 10.8 × 10^3^ (T5)]. A medical history for inflammatory CNS diseases was not reported by the patient and no clinical features beyond the flaccid amyotrophic proximal tetraparesis were noticed. Because white cell counts and lactate levels were normal, we classify the findings in this patient as immunological scar due to a former infection.

#### Qalb

Qalb was elevated in 14 SMA patients at baseline (T1). Qalb increased from baseline (T1) to the following six lumbar punctures [T1–T2 (*p* = 0.0093), T1–T3 (*p* < 0.0001), T1–T4 (*p* = 0.0001), T1–T5 (*p* = 0.0324), T1–T6 (*p* = 0.0002), T1–T7 (*p* = 0.0054)]. At T8 median Qalb levels were lower compared to baseline (T1) (*p* = 0.0469), however, only 8 patient samples could be evaluated at T8.

Since Qalb depends on age, Qalb values were further analyzed within three age subgroups. Median values with quartiles and ranges of Qalb age subgroups are presented in Table 3.

**Table 3:** Illustration of Qalb levels within age subgroups.

	**SMA patients** ***≤ 15 years*** **Mean age** **12.5 (SD 2.8)** **range 7.0–15.0** ***N* = 8**	**SMA patients** ***>15 and ≤ 40 years*** **Mean age** **27.1 (SD 7.0)** **range 16.0–39.0** ***N* = 33**	**SMA patients** ***>40 and ≤ 60 years*** **Mean age** **48.4 (SD 4.9)** **range 41.0–60.0** ***N* = 19**
Qalb × 10^3^ T1	4.00 [2.90; 4.85] range 2.20–11.40 *N* = 8	4.14 [3.40; 6.07] range 2.60–8.08 *N* = 30	4.69 [3.90; 7.20] range 2.26–10.40 *N* = 19
Qalb × 10^3^ T2	3.90 [3.10; 5.40] range 2.70–12.20 *N* = 7	4.35 [3.60; 5.85] range 2.30–11.50 *N* = 32	5.74 [4.35; 6.85] range 3.30–15.60 *N* = 17
Qalb × 10^3^ T3	4.50 [3.50; 4.90] range 3.40–11.00 *N* = 7	4.52 [3.79; 6.44] range 2.50–11.66 *N* = 28	5.06 [4.13; 7.23] range 2.19–14.34 *N* = 16
Qalb × 10^3^ T4	4.20 [3.60; 5.10] range 3.40–11.70 *N* = 7	4.39 [3.48; 6.98] range 2.60–10.30 *N* = 26	5.92 [4.16; 8.40] range 3.20–15.00 *N* = 17
Qalb × 10^3^ T5	[2.90; 4.70] range 2.80–10.80 *N* = 7	4.85 [3.63; 6.08] range 2.70–9.60 *N* = 22	5.05 [4.07; 7.20] range 2.14–11.20 *N* = 16
Qalb × 10^3^ T6	[3.25; 4.25] range 3.10–4.60 *N* = 5	5.46 [4.03; 6.37] range 2.70–9.68 *N* = 17	5.57 [4.30; 7.40] range 2.42–13.14 *N* = 15
Qalb × 10^3^ T7	2.50, 4.00, 4.20, 5.10 *N* = 4	5.16 [4.21; 6.76] range 3.10–12.48 *N* = 14	4.30 [3.60; 8.20] range 2.39–9.88 *N* = 7
Qalb × 10^3^ T8	3.50, 3.60, 4.00 *N* = 3	3.80, 5.90, 7.14, 7.81 *N* = 4	4.30 *N* = 1

*Median values with quartiles (in square brackets) and ranges of Qalb at treatment days T1–T8 in SMA patients undergoing treatment with nusinersen are shown. Qalb, albumin CSF/serum quotient; N, number of available samples*.

##### Age-related Qalb.

*SMA patients ≤ 15 years*. In the subgroup of patients ≤ 15 years, Qalb at baseline was only elevated in the previously reported 15-year-old SMA type 3 patient.

Qalb did not differ significantly from baseline (T1) to the following lumbar punctures in this subgroup [T1–T2 (*p* = 0.5000), T1–T3 (*p* = 0.0938), T1–T4 (*p* = 0.1563), T1–T5 (*p* = 1.0000), T1–T6 (*p* = 0.3750), T1–T7 (*p* = 0.1250), and T1–T8 (*p* = 0.500)].

*SMA patients between 16 and 40 years*. Qalb was slightly elevated in 8 patients of this subgroup at baseline (range 5.8–8.1 × 10^3^). Compared to baseline, Qalb increased significantly from baseline T1–T3 (*p* = 0.0025), T1–T4 (*p* = 0.0190), T1–T6 (*p* = 0.0032), and T1–T7 (*p* = 0.0398), but did not reach the level of significance at T1–T2 (*p* = 0.1602), T1–T5 (*p* = 0.2126), and T1–T8 (*p* = 0.2500).

*SMA patients between 41 and 60 years*. Qalb at baseline was elevated in 5 patients of this age group (range 7.2–10.4 × 10^3^).

Qalb increased significantly from baseline T1–T2 (*p* = 0.0461), T1–T3 (*p* = 0.0347), T1–T4 (*p* = 0.0214), and T1–T6 (*p* = 0.0492). No significant difference in Qalb levels could be observed from T1 to T5 (*p* = 0.1046) and from T1 to T7 (*p* = 0.8125). A comparison from T1 to T8 could not be made due to low sample size (only one sample).

#### Lactate

35 of 60 CSF baseline lactate values were under the lower reference level of 1.5 mmol/L. Two patients showed mild elevated lactate values above the reference level of 2.1 mmol/L at baseline and in following samples (patient 1 range: 2.0–3.1 mmol/L; patient 2 range: 2.8–3.1 mmol/L); since both patients have diabetes mellitus type 2 and obesity, we evaluate lactate levels in this context. In CSF of three patients, we found transient mildly increasing lactate levels during therapy (≤2.5 mmol/l).

Lactate levels showed a stable course during therapy and did not differ from T1 to T2 (*p* = 0.8445), T1 to T3 (*p* = 0.1760), T1 to T4 (*p* = 0.8240), T1 to T5 (*p* = 0.9421), T1 to T6 (*p* = 0.6803), T1 to T7 (*p* = 0.4954), and T1 to T8 (*p* = 0.1797).

#### OCB

OCB were analyzed in 51 of 60 patients at baseline. In 48 of these patients, OCB type 1 or 4 were present in baseline and in follow-up CSF/serum samples.

Two patients showed positive OCB in CSF (type 2 and 3) in all assessed samples. While in one of these patients concomitant high protein and Qalb levels were observed (described before), in the other, a 45-year-old SMA type 3 patient, further routine CSF parameter were all within the range. Currently or previously existing clinical symptoms indicating an inflammatory CNS disease were also not evident in this patient. Moreover, positive OCB in CSF (type 3) were observed in a 42-year-old SMA type 3 patient, however only at T3. The corresponding CSF and serum samples were re-examined, but positive OCB in CSF were still detectable. Besides slightly increased total protein and Qalb levels, all other routine CSF parameters were within the normal range (T1–T7).

### Associations

#### CSF Total Protein

No association was found between total protein at baseline (T1) and age (rho = 0.25, *p* = 0.0559), scoliosis (Rpb = 0.02, *p* = 0.8856), spinal instrumentation (rPB = −0.12, *p* = 0.3672), and HFMSE (rho = −0.01, *p* = 0.9117). However, an association was found between total protein at baseline and SMA type (2 and 3) (rPB = 0.28, *p* = 0.0341) with higher total protein levels in the SMA type 3 subgroup.

We found no association between changes of total protein levels during therapy (T1–T8) and age (rho = 0.11, *p* = 0.3878), scoliosis (rPB = 0.04, *p* = 0.7405), spinal instrumentation (rPB = −0.01, *p* = 0.9309), SMA type (rPB = 0.05, *p* = 0.6955), or HFMSE (rho = 0.08, *p* = 0.5572).

Associations of total protein and SMA type 1 or CHOP INTEND scores were not statistically analyzed due to low number of SMA type 1 patients.

#### Qalb

Apart from the association between Qalb at baseline (T1) and age (rho = 0.31, *p* = 0.0203), we detected an association between Qalb at baseline and SMA type (2 and 3) (rPB = 0.32, *p* = 0.0186) with higher Qalb levels in the SMA type 3 subgroup. Association between Qalb at baseline and scoliosis (rPB = −0.02, *p* = 0.8877), spinal instrumentation (rPB = −0.06, *p* = 0.6502), and HFMSE (rho = 0.03, *p* = 0.8459) were not significant.

We found no association between changes of Qalb levels during therapy (T1–T8) and age (rho = 0.12, *p* = 0.3591), scoliosis (rPB = 0.04, *p* = 0.7683), spinal instrumentation (rPB = 0.15, *p* = 0.2539), SMA type (rPB = −0.05, *p* = 0.7360), or HFMSE (rho = −0.12, *p* = 0.3862).

Associations of Qalb and SMA type 1 or CHOP INTEND scores were not statistically analyzed due to low number of SMA type 1 patients.

**Figure 1:**
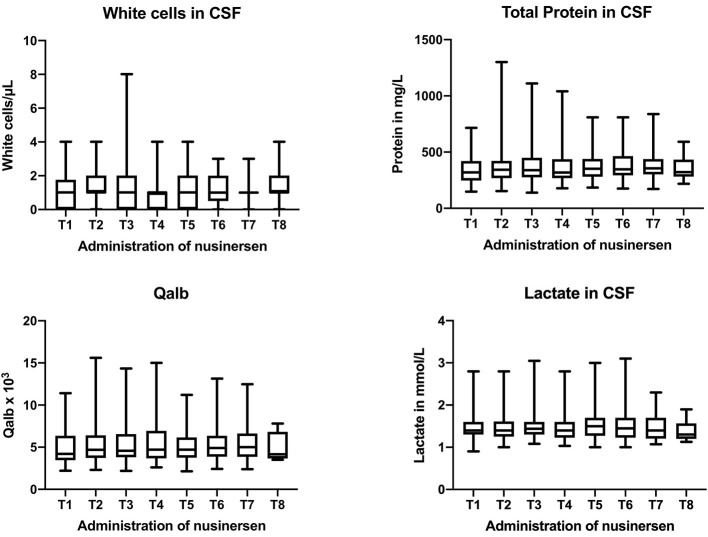
Boxplots of routine CSF parameters (white cells, total protein, Qalb, and lactate) at all treatment days (T1–T8). Boxes give median values with quartiles, whiskers indicate the range.

Boxplots of markers (white cells, total protein, Qalb, and lactate) are shown in Figure 1.

## Discussion

The present study evaluates routine CSF parameters from SMA patients treated with the ASO nusinersen.

Apart from a single white cell count increase of 8 cells/μL, there was no white cell count elevation above the normal range of 4/μL in all analyzed CSF samples. We classified the cell count increase in the context of a coincidental systemic and meningeal viral infection. Cases of meningitis under treatment with nusinersen including three cases of aseptic meningitis, which had been suggested as drug-induced, were reported in the past (6). Although the underlying pathophysiological mechanism of DIAM remains unclear, hypersensitivity reaction or direct irritation of the meninges during intrathecal administration of the drug are suggested. However, DIAM is a diagnosis of elimination and infectious causes have to be ruled out before (17). Repeated lumbar punctures may also lead to unspecific CSF pleocytosis, however, no further clinical symptoms would be expected. Interestingly, hemosiderophages were detected in the cytological examination of this sample in addition, although no (artificial) bleeding in CSF of T1 and T2 (erythrocytes 0/μL) had been observed. Hemosiderophages were also described in the fourth CSF sample (T4) of another patient without CSF pleocytosis; also, here, no signs of bleeding (erythrocytes 0/μL) had been found in previous CSF samples. Therefore, it can be assumed that repetitive lumbar punctures may lead to slight intraspinal bleeding even in atraumatic procedures. This may be important regarding the appearance of hydrocephalus, as bleedings into CSF space can cause arachnoid cell adhesions and thus disturbance of CSF resorption (18, 19). In these two patients, however, there is no clinical evidence for the development of a hydrocephalus so far.

Total protein showed an increase from baseline to the following treatment days except for treatment day T4. Qalb levels increased from baseline to the follow-up lumbar punctures and administrations of nusinersen. These results are in line with a previous study, where we observed slightly increased CSF total protein and Qalb levels after four injections of nusinersen (8) and are thus confirmed in a larger group of SMA patients and over a longer treatment period. Already in baseline CSF, some SMA patients presented with mildly elevated total protein and Qalb levels. Total protein and in particular Qalb can be used to evaluate BCB integrity and CSF flow, respectively (7, 20). The BCB is a functional barrier for the diffusion and filtration of macromolecules from blood to CSF and not be equated with the BBB, which represents a morphological barrier (21). Increased permeability of BCB capillaries has been considered to be the main pathophysiological mechanisms contributing to elevated CSF protein concentrations; but, the concept of a reduced CSF flow rate leading to increased protein levels in CSF is currently favored (7, 22). High CSF protein and Qalb concentrations are found in newborns, but decrease gradually during the first year of life, and are at low levels during childhood. In adults, CSF protein and Qalb levels increase with age (23, 24). Dysfunction of the BCB or CSF flow, respectively, is present in various neurological diseases of inflammatory and non-inflammatory etiology and can even be found in patients without manifest neurological disease (14). Elevated CSF protein and Qalb concentrations are typically found in infectious (25–28) and non-infectious (16, 29) inflammatory diseases of the CNS, in subarachnoidal hemorrhage and CNS neoplasm (30). Regarding the subgroup of motoneuron disease patients, elevated levels of CSF total protein and Qalb levels were described as well (13, 31). Moreover, CSF total protein and Qalb are influenced by body weight, sex, degenerative lower back disease, spinal stenosis, hypothyroidism, alcohol consumption, smoking, and physical activity (32–35).

Besides the association of Qalb levels at baseline with age, we found an association of total protein and Qalb at baseline with SMA type, but no association with scoliosis, spinal instrumentation or HFMSE. As clinical features vary widely between patients, especially in SMA type 3 (e.g., ambulatory vs. non-ambulatory), we speculate, that the association of baseline total protein and Qalb to SMA type is determined by age (as the SMA type 2 patients were younger than the SMA type 3 patients) (24), even if the association of age and total protein itself did not reach statistical significance (*p* = 0.0559). Regarding changes of total protein and Qalb levels during therapy, we found no association to the clinical features (age, scoliosis, spinal instrumentation, HFMSE, or SMA type). We suppose that the slight change in CSF total protein and Qalb is caused by repeated lumbar punctures and/or intrathecal administration of the drug and is not further influenced by the investigated clinical characteristics. Data on CSF findings in repeated lumbar punctures are difficult to compare because they are usually gathered in patients with other (CSF) pathologies (e.g., idiopathic intracranial hypertension) (36). It is possible that CSF flow slows down under therapy, but if there is a relation to the development of hydrocephalus (37) cannot be answered with our study. None of our patients showed clinical signs of developing hydrocephalus. But further data, such as CSF opening pressure and cerebral imaging would be helpful in approaching this question. Moreover, it is of note, that the appearance of communicating hydrocephalus under nusinersen therapy has been diagnosed mainly in very young patients so far (3 out of 5 patients younger than 1 year) (5); as already mentioned, BCB and CSF flow characteristics are changing within the first year of life (23). Thus, our observations of total protein and Qalb dynamics in SMA patients aged 11–60 years undergoing therapy may not automatically transferable to this patient group. Of note, total protein levels showed an increase from baseline to the following lumbar punctures, but total protein levels at all treatment days after baseline were at similar levels. Furthermore, we only investigated a period of 18 months of treatment in these patients, and samples size decreased over time.

Lactate values were stable over all lumbar punctures and administrations of nusinersen. More than 50% of the SMA patients had lactate levels below the lower reference boarder of 1.5 mmol/L in baseline CSF, but decreased lactate values are not indicative of specific pathologies. Only in three patients lactate level increased slightly above the reference boarder during therapy. Lactate levels in CSF can be increased in a variety of inflammatory, vascular, metabolic, and neoplastic diseases of the brain and meninges. In our patients however changes were marginal, so we consider them to be unspecific.

Transient positive OCB in CSF (type 3) were observed in a 42-year-old SMA type 3 patient. Oligoclonal intrathecal IgG antibody synthesis is not specific and there are in general several possibilities of interpretation: intrathecal IgG synthesis can be a sign of an acute disease with a monocausal reaction to a specific antigen, it can be a post-acute antibody synthesis (with subsequently decreasing intrathecal synthesis) without clinical significance or intrathecal antibody synthesis is part of a poly-specific immune response, as typically found in acute and especially chronic inflammatory diseases of the CNS (38). Since this patient showed no further signs of intrathecal inflammation in CSF (normal white cell count and lactate, slight total protein and Qalb elevation already in the baseline sample), a monocausal reaction to a specific antigen might be a possible explanation of oligoclonal IgG synthesis in CSF. ASOs are potentially immunogenic (39) and in plasma samples of SMA patients treated with nusinersen, anti-drug antibody (ADA) synthesis, including neutralizing antibody, have been reported. Thirteen patients (6% of the evaluated 229 patient plasma samples) developed treatment-emergent ADAs, of which 2 were observed transiently, 5 were considered to be persistent and 6 were unconfirmed. Although only limited data exist so far, ADAs are not expected to have an impact on clinical response, adverse events or the pharmacokinetic profile of nusinersen (6). Whether the OCB in CSF we observed in this patient may be correlates of intrathecal synthesized ADA, however, is currently highly speculative and needs further investigation.

In summary, the examination of CSF routine parameters in 60 SMA patients (types 1, 2, and 3, aged 7–60 years) treated with nusinersen revealed that repeated intrathecal administration of the drug seems to be in general safe and well-tolerated. This is in line with previous but limited results from clinical phase 1 studies on intrathecal delivery of ASOs in human (40–42). Nevertheless, since there is little overall experience with ASOs so far, it is important to record and report potential side effects [*Details of the national reporting systems to communicate adverse reactions (side effects) of drugs are available e.g., on the website of the authorities (e.g.*, *http://www.ema.europa.eu**) and pharmaceutical companies (e.g., Biogen)*]. Our data suggest that a systematic examination of routine CSF parameters in patients in which intrathecal ASOs are administered is important to obtain information on possible side effects and to gain further insights into intrathecal processes.

## Data Availability Statement

The datasets generated for this study will not be made publicly available as it concerns patient data.

## Ethics Statement

The studies involving human participants were reviewed and approved by University of Ulm approval number 19/12; 2012. Written informed consent to participate in this study was provided by the patients and/or patients' legal guardian/next of kin.

## Author Contributions

CW and RG designed the study. CW and JD performed statistical analysis. CW wrote the manuscript. All authors, were involved in the data collection process analyzed global study data and revised and approved the final version of the manuscript.

### Conflict of Interest

The authors declare the following potential conflicts of interest with respect to the research, authorship, and/or publication of this article: CW has received honoraria from Biogen as advisory board member and for lectures and as consultant from Hoffmann-La Roche. She also received travel expenses from Biogen. JK has received financial research support from TEVA Pharmaceuticals and honoraria as speaker/consultant for AbbVie, Allergan, Biogen, Ipsen, and AveXis/Novartis. MO received honoraria as consultant for Biogen, Axon, and Fujirebio. ZU has received honoraria from Biogen as consultant. BW has received honoraria from Biogen for a lecture. PW has received honoraria from Biogen and Desitin for lectures and as a consultant from Hoffmann-La Roche and Abbvie. AL received financial research support from AB Science, Biogen Idec, Cytokinetics, GSK, Orion Pharma, Novartis, TauRx Therapeutics Ltd. and TEVA Pharmaceuticals. He also has received honoraria as consultant from Mitsubishi, Orion Pharma, Novartis, Teva and as advisory board member of Biogen, Treeway, and Hoffmann-La Roche. MD received a travel grant from Biogen and speaker honoraria from Desitin and Genzyme. PL has received financial research support from TEVA Pharmaceuticals and honoraria as speaker/consultant for AbbVie, BIAL, Desitin,Licher MT, Medtronic, Novartis. HT has received research support and/or honoraria as speaker/consultant/advisory board member from Bayer, Biogen, Celgene, Fresenius, Genzyme Sanofi, Merck, Mylan, Novartis, Roche Diagnostics, Teva. AH has received honoraria from Biogen and Desitin as advisory board member. RG has received honoraria from Biogen as advisory board member. The remaining authors declare that the research was conducted in the absence of any commercial or financial relationships that could be construed as a potential conflict of interest.
